# Neonatal Astrocyte Damage Is Sufficient to Trigger Progressive Striatal Degeneration in a Rat Model of Glutaric Acidemia-I

**DOI:** 10.1371/journal.pone.0020831

**Published:** 2011-06-15

**Authors:** Silvia Olivera-Bravo, Anabel Fernández, María Noel Sarlabós, Juan Carlos Rosillo, Gabriela Casanova, Marcie Jiménez, Luis Barbeito

**Affiliations:** 1 Cellular and Molecular Neurobiology Department, Instituto Clemente Estable, Montevideo, Uruguay; 2 Comparative Neuroanatomy Associated Unit of the School of Sciences, Cellular and Molecular Neurophysiology Department, Instituto Clemente Estable, Universidad de la Republica, Montevideo, Uruguay; 3 Electron Microscopy Unit, Comparative Neuroanatomy Associated Unit of the School of Sciences, Universidad de la Republica, Montevideo, Uruguay; 4 Institut Pasteur Montevideo, Montevideo, Uruguay; Federal University of Rio de Janeiro, Brazil

## Abstract

**Background:**

We have investigated whether an acute metabolic damage to astrocytes during the neonatal period may critically disrupt subsequent brain development, leading to neurodevelopmental disorders. Astrocytes are vulnerable to glutaric acid (GA), a dicarboxylic acid that accumulates in millimolar concentrations in Glutaric Acidemia I (GA-I), an inherited neurometabolic childhood disease characterized by degeneration of striatal neurons. While GA induces astrocyte mitochondrial dysfunction, oxidative stress and subsequent increased proliferation, it is presently unknown whether such astrocytic dysfunction is sufficient to trigger striatal neuronal loss.

**Methodology/Principal Findings:**

A single intracerebroventricular dose of GA was administered to rat pups at postnatal day 0 (P0) to induce an acute, transient rise of GA levels in the central nervous system (CNS). GA administration potently elicited proliferation of astrocytes expressing S100β followed by GFAP astrocytosis and nitrotyrosine staining lasting until P45. Remarkably, GA did not induce acute neuronal loss assessed by FluoroJade C and NeuN cell count. Instead, neuronal death appeared several days after GA treatment and progressively increased until P45, suggesting a delayed onset of striatal degeneration. The axonal bundles perforating the striatum were disorganized following GA administration. In cell cultures, GA did not affect survival of either striatal astrocytes or neurons, even at high concentrations. However, astrocytes activated by a short exposure to GA caused neuronal death through the production of soluble factors. Iron porphyrin antioxidants prevented GA-induced astrocyte proliferation and striatal degeneration in vivo, as well as astrocyte-mediated neuronal loss in vitro.

**Conclusions/Significance:**

Taken together, these results indicate that a transient metabolic insult with GA induces long lasting phenotypic changes in astrocytes that cause them to promote striatal neuronal death. Pharmacological protection of astrocytes with antioxidants during encephalopatic crisis may prevent astrocyte dysfunction and the ineluctable progression of disease in children with GA-I.

## Introduction

During CNS development, astrocytes are mostly generated after the initial production of neurons and then play a key role in the subsequent development of grey and white matter. Astrocytes participate in guiding the migration of developing axons and neuroblasts [1], [2], are essential for the generation and pruning of synapses [3], and for the blood brain barrier formation [4]. Developing astrocytes are vulnerable to ischemia [5], oxidative stress [6], and inflammation [7], [8]. Primary or secondary astrocyte damage has been implicated in several developmental or perinatal CNS pathologies such as periventricular leukomalasia [6], vanishing white matter disease [9], Alexander disease and lead and methylmercury poisoning [9]. Thus, a vulnerability of astrocytes in early stages of development may critically alter subsequent survival and function of neurons.

Severe loss of basal ganglia neurons is a pathological hallmark of Glutaric acidemia type I (GA-I), an autosomal recessive inherited neurometabolic disease caused by deficiency of glutarylCoA dehydrogenase (GCDH) enzyme [10], [11], [12], [13]. GCDH deficiency alters lysine and tryptophan catabolism causing the accumulation of glutaric acid (GA) and related metabolites in the brain of GA-I patients [10], [14]. Clinically, babies with GA-I can present macrocephaly before the appearance of first symptoms typically denoted by encephalopathic crisis [11], [15], [16]. Then, GA-I may evolve to a complex neurological syndrome simulating a cerebral palsy with extrapyramidal signs such as progressive dystonia and dyskinesia. Symptoms may have a gradual rate of onset and progression, or occur suddenly after an acute metabolic crisis [10], [11], [12], [16], [17]. Pathologically, the characteristic features of GA-I are a loss of neurons in the caudate and putamen and spongiform lesions in the white matter [11], [15], [18]. Increased extracellular GA acts as a potent neurotoxic metabolite having the potential to induce excitotoxicity [17], disruption of mitochondrial energy metabolism and oxidative stress [19], [20]. In astrocytes, GA interferes with sodium-coupled dicarboxylate transporters, thus disrupting the supply of tricarboxylic acid cycle intermediates necessary for ATP and neurotransmitter synthesis in neurons [21]. In spite of this acute metabolic effect, there is scarce information about other mechanisms by which GA may cause astrocytes to trigger progressive neuronal loss in GA-I.

We have previously shown that astrocytes are preferential cell targets of GA [22] which likely accumulates in astrocytes through glutamate transporters [23]. Remarkably, cultured astrocytes become severely dysfunctional when exposed to GA, with mitochondrial depolarization and secondary oxidative stress [22]. In addition, GA induces astrocytes to actively proliferate by a mechanism involving activation of MAP kinases and oxidative stress. We have also showed that systemic administration of GA to rat pups also resulted in acute increase in postnatal gliogenesis and increased number of undifferentiated astrocytes expressing S100β [22]. However, it is uncertain whether the appearance of such abnormal astrocytes contributes to the striatal degeneration characteristic of the disease.

To investigate the role of astrocytes in GA-I striatal degeneration, a transient metabolic crisis was induced in rat pups by a single intracerebroventricular (icv) administration of GA to mimic an acute encephalopathic crisis suffered by GA-I patients [12], [14], [19]. Here, we describe a novel mechanism by which icv GA acutely induced proliferation of astrocytes and long-term astrocytosis. Interestingly, astrocytosis induced by GA was followed by massive neuronal loss days after the crisis indicating an indirect mechanism of toxicity. In culture systems, GA was not directly toxic to isolated striatal neurons, but caused oxidative stress and long lasting astrocyte dysfunction sufficient to kill striatal neurons. These results indicate that dysfunctional astrocytes are sufficient to trigger striatal neuronal loss, thus providing the basis to prevent the progressive neurodegeneration using antioxidants.

## Results

### GA induced long lasting astrocytosis in the striatum

**Figure 1:**
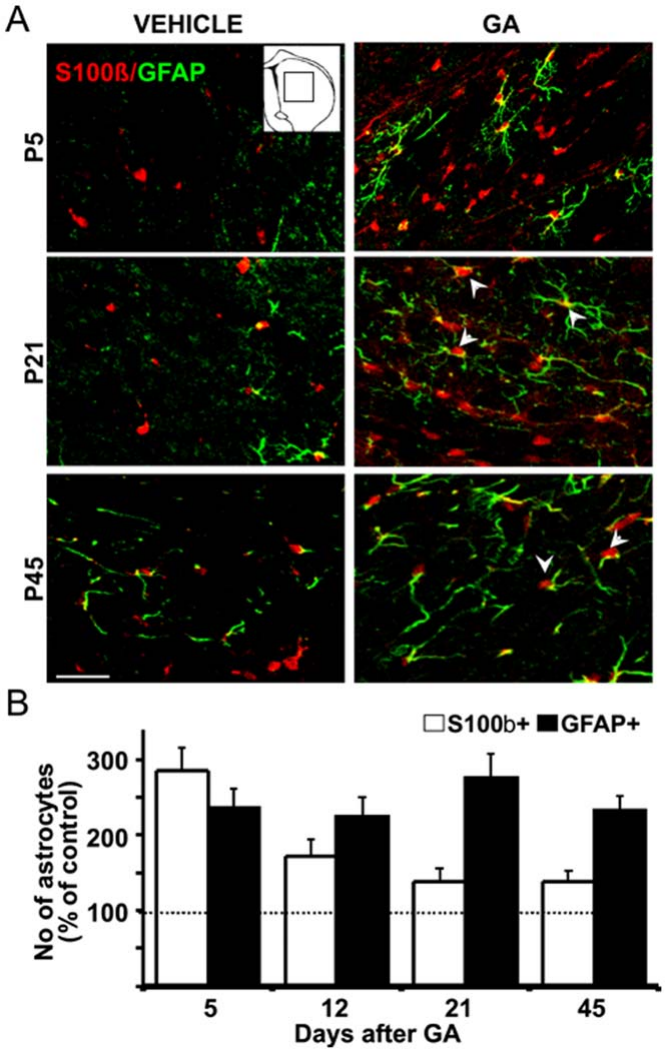
GA induced long lasting astrocytosis in the striatum. P0 rat pups were injected icv with GA or vehicle and processed from 5 (P5) until 45 (P45) days later for immunohistochemistry as described in Materials and Methods. **A:** Representative S100β (red) and GFAP (green) immunofluorescences of the striatal parenchyma at P5, P21 and P45 evidencing an increased number of astrocytes with nuclear S100β and typical GFAP stains in GA-treated pups (right) when compared to controls (vehicle, left). Some GFAP astrocytes were also positive to S100β (white arrow heads). Box in the inset shows striatal areas analyzed. Scale bar: 25 µm. **B:** Increased number of astrocytes after the icv GA injection evidenced by 2–3 folds rises in S100β positive cells at P5 and elevated number of GFAP positive cells until P45. All values were significantly higher (p<0.05) than respective controls (taken as 100% and indicated with the dotted line). All data is expressed as mean ± SEM.

In line with a previous report [22], we have validated an animal model of GA-I by injecting rat pups at postnatal day 0 (P0) with a single bolus of 2.5 µmol/g body weight GA into the cisterna magna (IV ventricle). The dose employed likely reach millimolar concentration of GA in the brains of the pups [19], which correspond to the concentrations found in patients with GA-I [11], [12], [14], [19]. Furthermore, the dose was adjusted to also reproduce the characteristic encephalopatic crisis of GA-I patients. In pups, the crisis consisted in tonic-clonic convulsions lasting up to 15 min followed by a hypotonic phase that lasted up to 30 min. In average, there was a mortality of 20%. As depicted in Fig. 1, the pathological correlate of GA administration was a long lasting astrocytosis observed from P5 to P45. GA induced a 3-fold increase of astrocyte-like cells expressing nuclear S100β in P5 as compared to the respective age-matched controls injected with vehicle. Increased number of S100β positive cells remained elevated until P45. The number of GFAP astrocytes remained elevated by 2–3 folds from P5 to P45. Double labeled cells to both S100β and GFAP were increased from a 25%±7 at P5 up to 92%±18 at P45. No significant changes in striatal vimentin and nestin expression were found in GA-injected animals at all ages studied.

### Increased gliogenesis induced by GA

**Figure 2:**
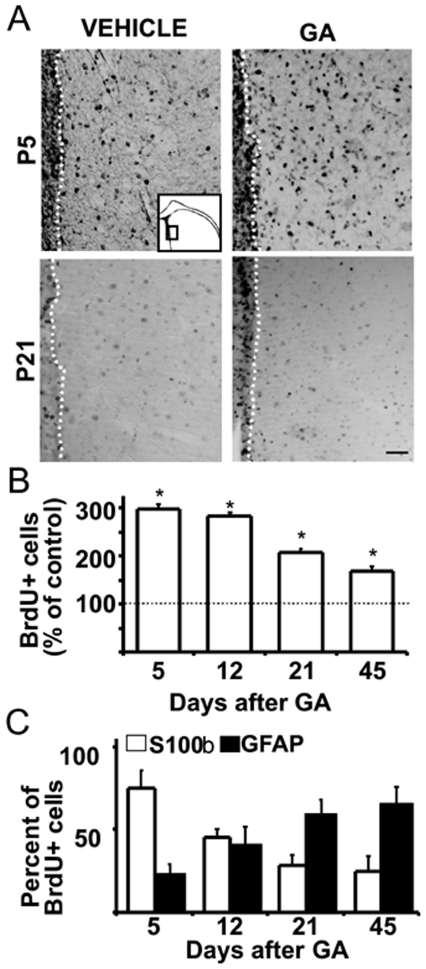
Increased gliogenesis following icv GA administration. P0 pups were treated with GA or vehicle and immediately injected with a single dose of BrdU ip to label dividing cells. **A:** Panoramic BrdU immunohistochemistry of the striatal regions lining ventricles showing an increased number of dark BrdU labeled nuclei in both striatal parenchyma and subventricular areas (dotted white lines indicate the limit between both areas) of GA-injected animals. In controls, injected with vehicle, BrdU positive nuclei were mainly restricted to subventricular regions. Inset schematizes areas imaged. Calibration bar: 75 µm. **B:** The number of BrdU labeled cells in the striatal parenchyma of GA-injected animals related to respective controls remained elevated until P45. All values were significantly higher than controls (taken as 100% and indicated by a dotted line). **C:** Astroglial phenotype of BrdU+ cells in GA-injected animals evidenced by a double labeling for both BrdU and S100β that were dominant at P5, and a progressive BrdU-GFAP double immunoreactivity at P21 and P45. In all conditions, the number of BrdU positive cells that displayed astroglial phenotype was significantly higher than controls. All data is expressed as mean ± SEM. Asterisks indicate statistical significance related to respective controls at p<0.05).

A single icv administration of GA at P0 induced a marked increase in dividing cells labeled with BrdU that were mainly localized in the striatal neuroepithelium and the underlying parenchyma (Fig. 2A, B). The number of BrdU positive cells in GA-treated pups striatal parenchyma remained elevated up to 45 days, suggesting the majority of newborn cells survived. Most of BrdU positive cells displayed an astrocytic phenotype predominantly expressing S100β at P5 but progressively turning to GFAP positive as animal aged (Fig. 2). In comparison, animals injected with vehicle displayed a low number of BrdU positive cells and few of them were labeled with S100β or GFAP.

### Delayed neuronal death following icv administration of GA

**Figure 3:**
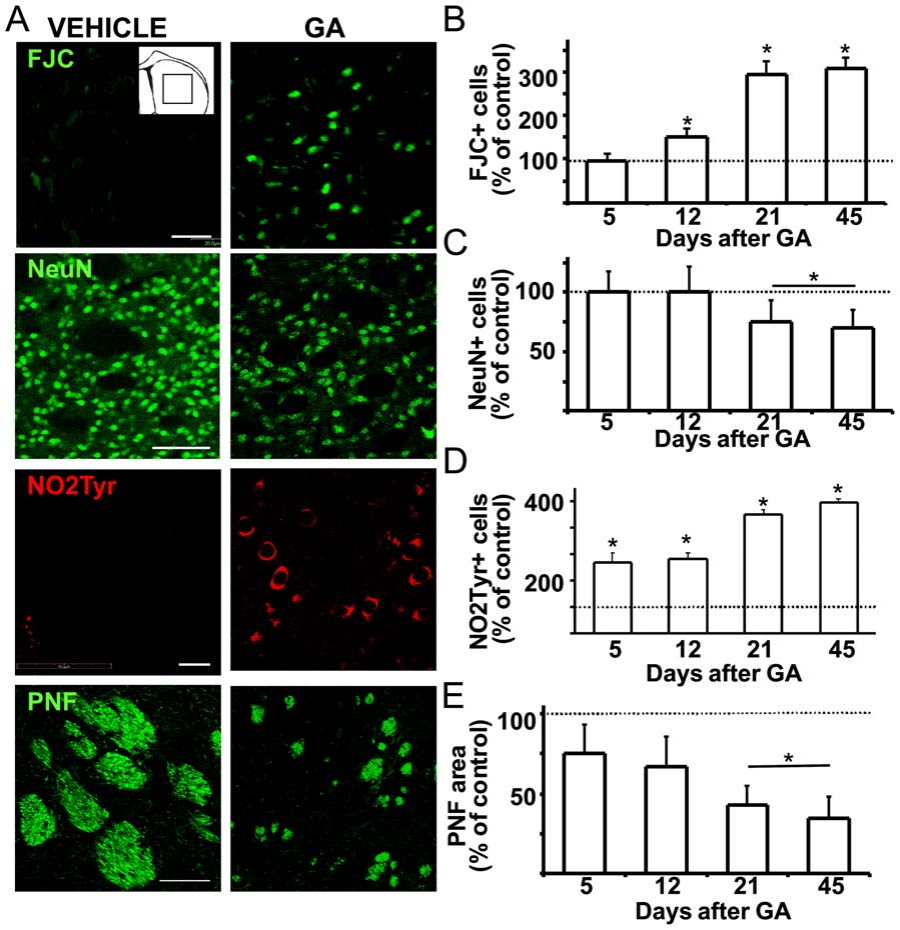
Delayed degeneration of striatal neurons following icv GA administration. P0 pups were treated with GA or vehicle and processed at different times to evaluate Fluoro JadeC (FJC) positive degenerating neurons, neuron number, nitrotyrosine immunoreactivity and phosphorylated neurofilament (PNF) areas. **A:** Representative microphotographs of the striatal parenchyma at P21 showing that GA treatment caused increased FJC and nitrotyrosine labeling, and both decreased number of NeuN positive neurons and PNF areas. Calibration: 20 µm in upper and mid panels, and 100 µm in the bottom one. Inset: scheme of striatal area analyzed. **B:** Time course of FJC labeling in striatal sections of GA-injected animals as compared to controls. Note the sharp increase in degenerating FJC positive cells at P21 and P45. **C:** Time course of nitrotyrosine labeling in striatal sections of GA-injected animals as compared to controls. Note the sharp increase in nitrotyrosine immunoreactivity at all ages but peaking at P21. **D:** Time course of number of striatal NeuN positive neurons showing a significant decrease at P21 and P45 in GA-injected animals as compared to controls. **E:** Relative area of PNF bundles at different times after GA-treatment. Note the significant diminution evidenced at P5 and the progressive decrease until P45. Dotted lines in charts B, C and D indicate respective control values. All data are the mean ± SEM. Statistical significance at p<0.05 (*).

To determine whether GA administration induced neurodegeneration, the number of degenerating striatal neurons was estimated by Fluoro JadeC (FJC) cell staining at different time points (Fig. 3A). While no significant number of degenerating neurons was found in control animals, the number of FJC degenerating neurons in GA-injected pups increased just after P12, reaching a 3-fold increase at P21 and P45 (Fig. 3B).

The striata of GA-injected animals also showed an increased number of neurons stained with nitrotyrosine (second row Fig. 3A, Fig. 3C), indicative of nitrosative and oxidative stress [6], [24]. Some oligodendrocytes inside axonal bundles were also stained for nitrotyrosine (not shown).

The number of striatal neurons (estimated by NeuN positive cell counting per area unit) remained unchanged until P12 in GA injected pups as compared to controls (Fig. 3D). This was followed by a significant decrease by 25 and 27% in NeuN positive neurons at P21 and P45, respectively. In addition, the pattern of NeuN neuronal staining in GA-injected animals was atypical, with a weak, perinuclear and cytoplasmic staining that contrasted with predominately nuclear staining in control pups (Fig. 3A, third row panels). As dying neurons could retain some NeuN immunoreactivity [25], the counting likely overestimates the total number of neurons.

The axonal bundles perforating the striatum were disrupted in GA-injected animals as shown by phosphorylated neurofilament (PNF) staining. While axonal bundles in control pups were progressively larger and compacted as the pup aged, bundles remained disaggregated in GA-injected animals, the total area being decreased by 70% at P45 (Fig. 3E). Similar results were obtained with non-phosphorylated neurofilament staining (not shown). Compared to control animals, the area of PNF-axonal bundles was significantly decreased from P5 to P45.

### FeTCPP abolished the delayed astrocytosis and striatal degeneration induced by GA

**Figure 4:**
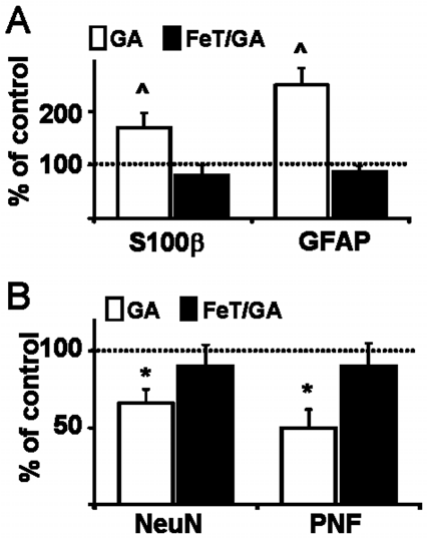
FeTCPP prevented GA-induced astrocytosis and subsequent striatal degeneration. P0 pups received a single dose of FeTCPP (20 µmol/g, ip) or vehicle and then were injected with GA or vehicle as described in Methods. **A:** Counting of S100β and GFAP astrocytes at P21. Note FeTCPP prevented astrocytosis induced by GA. ∧: p<0.01 related to controls. **B:** Counting of number of NeuN positive neurons and area of PNF bundles. FeTCPP co-administration with GA preserved both the neuron number and PNF area. Dotted lines indicate control value. *: p<0.05 related to corresponding controls.

The co-administration of FeTCPP intraperitoneal (20 nmol/g, ip) in pups receiving icv GA at P0 significantly prevented the increase in S100β and GFAP positive cells, neuronal loss and disruption of axonal bundles induced by GA (Fig. 4A–B), further indicating an association between oxidative damage acutely induced by GA [22] and subsequent astrocytosis and neuronal damage (Fig. 4 A–B).

### GA caused astrocytic dysfunction in vitro

**Figure 5:**
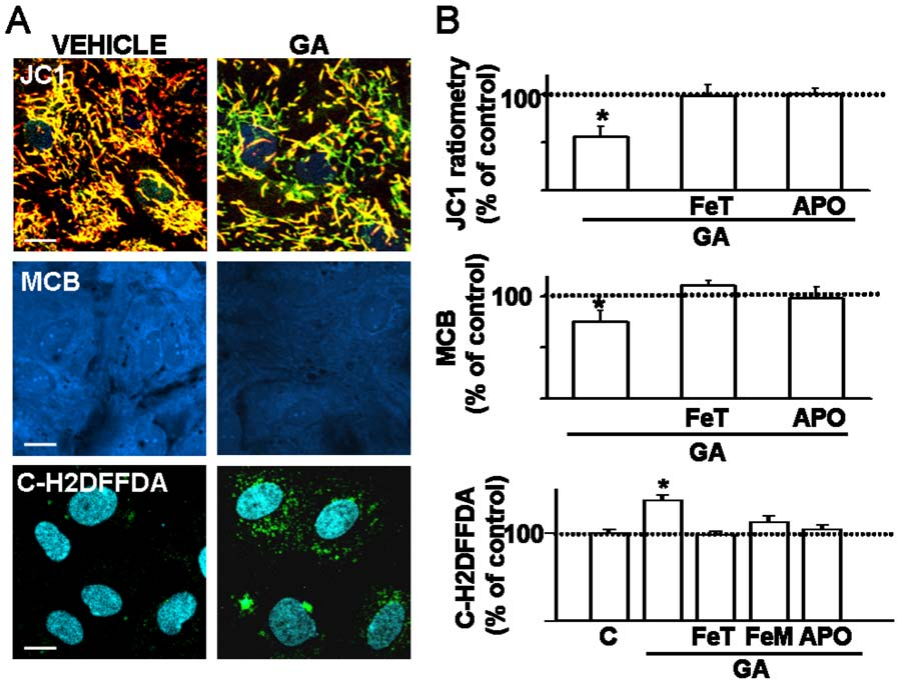
GA induced astrocytic mitochondrial depolarization and increased oxidative stress. Confluent striatal cultured astrocytes were submitted to GA (5 mM, 24 h) and then mitochondrial potential and oxidative status were assessed immediately. **A:** Representative images of the effects of GA on mitochondrial potential (measured by the probe JC1), glutathione levels (assessed by monochlorobimane), and oxidative activity analyzed with carboxy-H2DFFDA. Note that GA-treated astrocytes have decreased mitochondrial potential and cellular glutathione as evidenced by green mitochondria and less blue fluorescence, respectively. In bottom images, green spots surrounding the DAPI stained nuclei in GA-treated cells denote increased oxidative stress. Calibration: 20 µm in upper and mid panels, and 10 µm in the bottom one. **B:** Quantitation of GA effects and counteracting antioxidant actions. Charts show percent values of JC1 ratiometric fluorescence, monochlorobimane (MCB) emission and green carboxy-H2DFFDA fluorescence in GA-treated astrocytes alone or pre-incubated with the antioxidants FeTCPP (FeT, 20 µM), apocynin (APO, 1 mM), or FeTMPyP (FeM, 2 µM). Note that antioxidants abrogated GA decreasing effects on mitochondrial potential and glutathione levels, as well as the increased oxidative stress. Control conditions were indicated with dotted black lines. Asterisks indicate statistical significance at p<0.05.

Treatment of confluent astrocytes with 5 mM GA (24 h) caused significant mitochondrial depolarization as shown by decreased JC1 red staining and ratiometric red/green relationship (Fig. 5 A, B upper panel and chart). GA also evoked lower glutathione levels as indicated by decreased blue fluorescence resulting from monochlorobimane-glutathione adducts (Fig. 5 A, B middle images and graph). In addition, the oxidation of carboxy-H2DFFDA increased the green fluorescence up to 140% of controls in GA-treated astrocytes (Fig. 5 A, B bottom images and chart), further indicating oxidative stress. The antioxidants FeTCPP and apocynin [26] abrogated GA effects on cultured astrocytes (Fig. 5 B). GA did not affect immunoreactivity and expression of the astrocytic markers glutamate transporter GLT1 and glutamine synthase (not shown).

### GA did not induce neuronal death in absence of astrocytes

**Figure 6:**
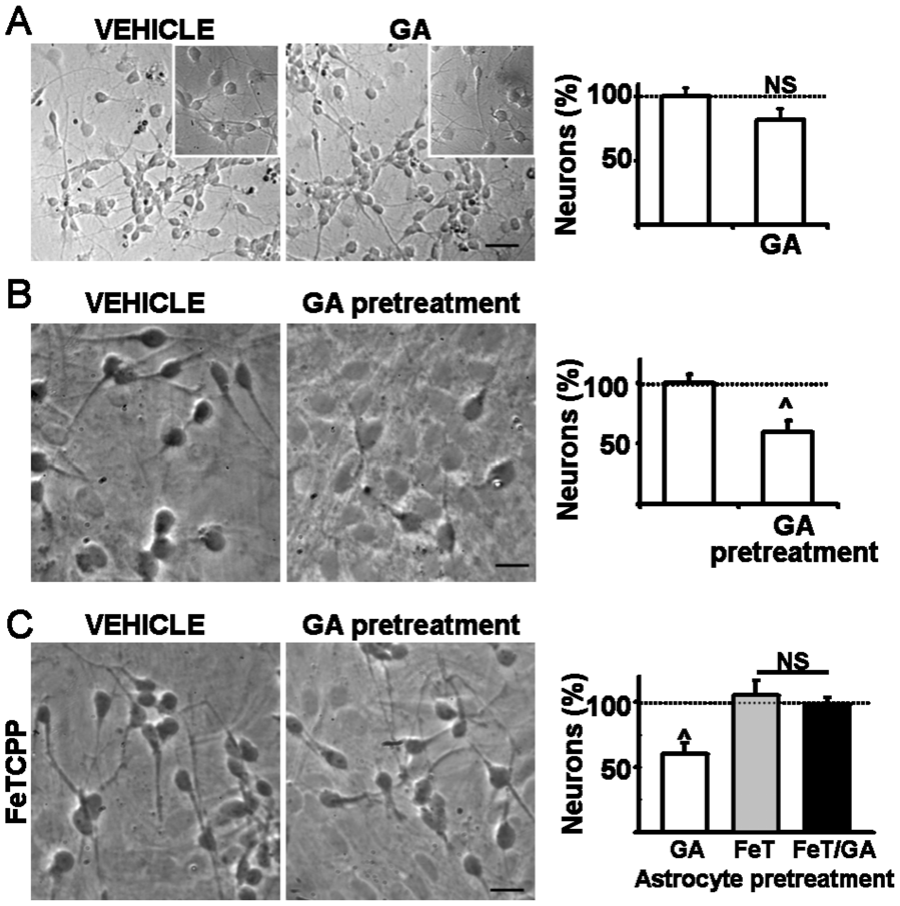
GA did not induce neuronal death in absence of astrocytes. Isolated striatal neuronal cultures were employed to investigate the mechanism of GA-induced neurotoxicity. **A:** Phase contrast of E18 striatal neurons at 5 DIV exposed to vehicle or 5 mM GA (pH 7.4, 24 h). Note that GA neither modified the morphology (as shown in respective insets) nor the number of MAP2 positive neurons as indicated in the right chart. Scale bars: 80 µm (images), 40 µm (insets). **B:** Astrocyte monolayers pretreated with vehicle or GA 5 mM for 24 h were used as feeding layer to cultured E18 striatal neurons. Note the decrease in the number of neurons and that surviving neurons exhibited swollen bodies and sparse neurites. Extensive washings before neuronal seeding discarded a direct effect of GA on striatal neurons. Scale bars: 20 µm. **C:** FeTCPP applied immediately before GA prevented toxic effects of GA-treated astrocytes as shown by the preserved neuronal number and morphology of neurons co-cultured on GA-treated astrocyte monolayers. Symbol ∧ indicates statistical significance at p<0.01 and NS: means no statistical signification.

Then, we used cell culture approaches to determine whether astrocytes were required for striatal neuron loss induced by GA. The direct neurotoxic potential of GA was assayed by incubating 5 days in vitro E18 striatal neurons with 5 mM GA (pH 7.4) or saline during 24 h. As shown in Fig. 6A, GA failed to induce significant neuronal death as estimated by counting of surviving neurons once the 24 h GA treatment finished. Lactate dehydrogenase release and number of MAP2 positive cells tested in GA treated neurons did not differ from controls (109%±12 and 89%±12, respectively), indicating a low vulnerability of isolated neurons to GA. This was consistent with a preserved neuronal morphology of GA-treated neurons (insets, Fig. 6A).

### Astrocyte activation by GA was sufficient to induce neuronal death

It was previously shown that GA activated astrocytes and induced proliferation [22]. When E18 striatal neurons were seeded on the top of a feeder layer of striatal astrocytes previously exposed to GA, the neuronal survival decreased by 50% as compared to those treated with vehicle only (Fig. 6B). Surviving neurons roughly lost up to 40% of processes and swollen its bodies around 25% when compared to control neurons (Fig. 6B).

Simultaneous treatment of astrocytes with GA and FeTCPP prevented astrocyte activation and subsequent neuronal death, and also preserved the normal neuronal morphology (Fig. 6C). Treating astrocytes with GA plus the antioxidants iron tetrakis-(4-sulfonatophenyl)-porphyrinate (FeTPPS, 20 µM) or iron tetrakis(N-methyl-4′-pyridyl)-porphyrinate (FeTMPyP, 2 µM) was also neuroprotective as shown by the obtained 95%±12 and 101%±8 of neuron survival, respectively. The NADPH oxidase inhibitor apocynin [26] also showed a modest protective effect (1 mM, 85%±15 of surviving neurons). 20 µM of the MAP kinase inhibitor 1,4-diamino-2,3-dicyano-1,4-bis [2-aminophenylthio] butadiene (U0126) partially prevented the neuronal loss induced by GA-stimulated astrocytes (70%±18 of neuron survival related to controls).

**Figure 7:**
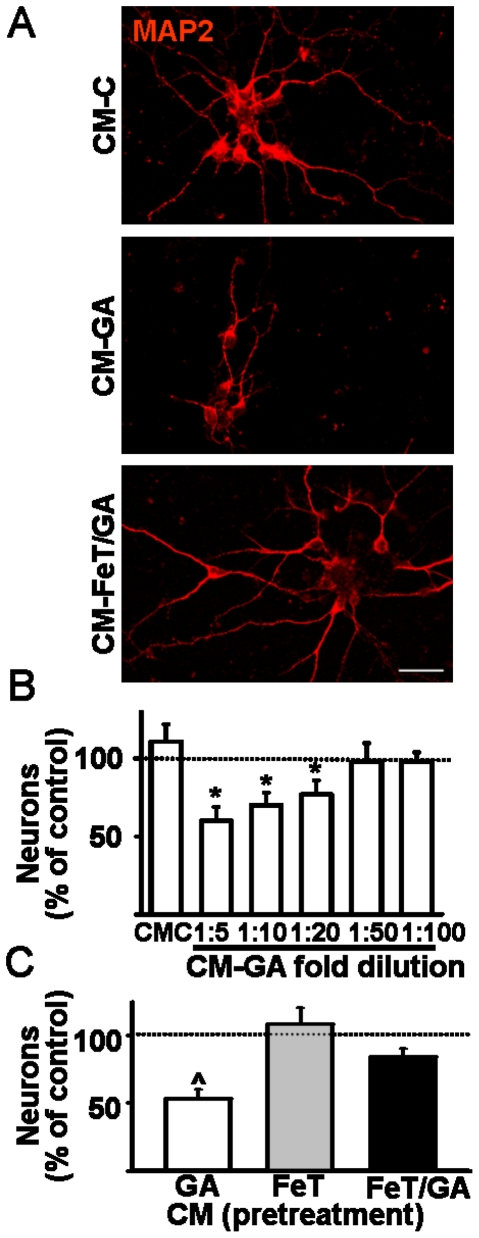
Astrocyte activation by GA was sufficient to induce neuronal death through soluble factors. The neurotoxic activity of conditioned media (CM) of astrocytes treated with GA (CM-GA) was tested on cultured E18 striatal neurons 5 DIV after plating. **A:** Representative MAP-2 immunostaining of striatal neurons upon treatment with CM from control (CM-C), or GA-treated (CM-GA) or FeTCPP/GA-treated astrocytes (CM-FeT/GA). Note decreased neuronal number and neurite growth caused by CM-GA and the maintenance of both neuronal survival and phenotype in the CM-FeT/GA condition. Scale bar: 50 µm. **B:** Counting of striatal neurons maintained in culture for 24 h with CM-C or increasing dilutions of CM-GA or vehicle. Neuronal number increased when treated with CM-C (first column of the chart). Conversely a concentration dependent neuronal loss was caused by CM-GA when compared with vehicle (taken as 100%, dotted line). *: p<0.05. **C:** FeTCPP applied immediately before GA abolished the toxicity of CM-GA (1∶5 dilution) preserving both the neuronal number and morphology. Control was indicated by the dotted line. All data is the mean ± SEM. ∧: p<0.01; *: p<0.05.

To determine whether astrocytes exposed to GA were sufficient to mediate neuronal death, we assessed the effects of astrocyte conditioned media (CM) on striatal neuron morphology and survival. 1∶5 dilution of CM obtained from control astrocytes (CM-C) exerted a beneficial effect on striatal neurons, increasing neurite growth and survival by 10% (Fig. 7A). In contrast, CM from GA-treated astrocytes (CM-GA) significantly decreased both neuronal survival and the number of neurites in surviving neurons. Maximal neuron death induced by CM-GA (approximately 45%) was obtained at a 1∶5 dilution but significant neuronal loss was seen up to 1∶20 dilution (Fig. 7B). Remarkably, neurotoxic potential of astrocytes treated with GA persisted up to 7 days after GA withdrawal. At this time, 1∶5 dilution of CM-GA killed 25%±10 of neurons, suggesting GA induced long-lasting astrocytic changes. Treatment of astrocytes with FeTCPP before the GA exposure prevented the toxicity of CM-GA (Fig. 7C), further suggesting oxidative stress is required for GA-induced astrocyte activation.

## Discussion

The present study shows evidence that astrocyte dysfunction induced by an acute and transient metabolic insult with GA is sufficient to initiate a progressive pathological process in astrocytes leading to striatal neuronal death. Neuronal degeneration was delayed by several days with respect to the metabolic insult and coincident with increased number of S100β-expressing astrocytes infiltrating the striatum. In addition, cultured astrocytes exposed to GA become no longer permissive to striatal neurons and secrete neurotoxic soluble factors, further indicating dysfunctional astrocytes are sufficient to initiate neuronal loss in GA-I. Taking together, these findings show a previously unknown pathological role of astrocytes in the triggering of striatal degeneration during postnatal development.

We found that a single icv administration of GA was sufficient to induce an acute encephalopatic crisis in rat pups, resembling that observed in patients with GA-I [15], with tonic-clonic convulsions followed by a progressive neurodegeneration. This effect was obtained by GA reaching transiently millimolar concentration in the pup's brain comparable to that found in the autopsy brains of patients [11], [12], [14], [15], [19]. Interestingly, GA administration did not result in acute or subacute striatal neuronal loss as could be anticipated if GA would simply act as a potent excitotoxin to induce neuronal death [17]. Rather, GA acutely stimulated striatal gliogenesis, which originated waves of newborn astrocytes that apparently remained integrated in the striatal parenchyma for several weeks. Such astrocyte population was predominantly S100β-positive during the first week and evolved to a predominant GFAP-positive astrocytosis lasting several weeks. This, in agreement with previous studies showing postnatal gliogenesis in the striatum is increased following brain damage such as hypoxia [27]. These results indicate that GA targets astrocytes more readily than previously thought and long before striatal neuronal damage is apparent.

This novel GA-I animal model allowed determining that astrocytes are affected early after GA administration, displaying a strong proliferative reaction and expressing increased levels of S100β [22]. GA is a dicarboxylic acid structurally close to glutamate that interacts with glutamate transporters [23] allowing GA accumulation in astrocytes preferentially. In turn, intracellular accumulation of GA in astrocytes results in severe mitochondrial dysfunction associated to oxidative stress and increased astrocyte proliferation via activation of MAPK signaling [22]. These results were further confirmed by a recent study showing GA competitively interferes with astrocytic sodium-dependent dicarboxylate transporters, altering its anaplerotic supply of tricarboxylic acid cycle intermediates to neurons [21]. Here, we provide evidence that dysfunctional astrocytes actively mediate the elimination of neurons in vivo and in vitro. In pups, neuronal degeneration was delayed by several days with respect to the metabolic insult and coincident with increased number of S100β-expressing astrocytes infiltrating the striatum. In addition, cultured astrocytes exposed to GA become no longer permissive to striatal neurons and secrete neurotoxic soluble factors,. Taking together, these findings show a previously unknown pathological role of astrocytes in the triggering of striatal degeneration during postnatal development.

Since astrocytosis was so blatant after GA administration we hypothesized that dysfunctional astrocytes are the cause of progressive neuronal death. A failure of astrocytes to reach appropriate differentiation may critically compromise the astrocytic support to neurotransmission or neuronal survival, as has been suggested in GA-I [21]. Alternatively, altered glutamate uptake by astrocyte could facilitate excitotoxic killing [2], [3], [4], [17], [28], [29]. Aberrant specification of glial precursors leading to an increased immature astrogliogenesis has also been proposed as a mechanism for abnormal neuronal organization and survival in postnatal development [28]. In addition, an exacerbated proliferation of astrocytes could impair the generation of oligodendrocytes from common glial precursors [28], contributing to white matter defects described in GA-I patients [10], [11]. Since S100β protein is typically secreted by astrocytes, its GA-induced expression could explain astrocyte proliferation and abnormal phenotypes. S100β is known to exert paracrine effects that contribute to astrocyte proliferation, migration, differentiation and neuronal death [30], [31]. Taken together, our results suggest that dysfunctional astrocytes are generated in response to GA administration and persist several weeks, being temporally coincident to the triggering of neuronal death. GA-induced delayed neurodegeneration seems to be a progressive process that occurs autonomously from GA levels, resembling some cases of GA-I patients where neurological symptoms continue to aggravate after normalization of metabolic parameters by dietary managements [11], [12], [13].

In the present study, we found that GA caused astrocytes to become neurotoxic for striatal neurons plated on top of highly enriched astrocyte monolayers. It is unlikely this effect was mediated by GA or related metabolites, since extensive washing was performed before neuronal plating. Astrocyte-mediated toxicity was further confirmed in experiments using the conditioned media of astrocytes following GA exposure, suggesting a mechanism mediated mainly by soluble factors. The mechanism of astrocyte-mediated toxicity to striatal neurons is under active investigation. It is unlikely that astrocytes kill neurons simply by decreasing the export of tricarboxylic acid cycle intermediates [21], since this mechanism is potentially reversible once GA is removed from the cultures or from the brain. Rather, we propose GA induces a long term phenotypic changes in astrocytes that causes them to become neurotoxic. Early oxidative stress, mitochondrial dysfunction and increased proliferation appear as crucial pathways contributing to astrocyte toxicity as suggested by the potent neuroprotective effects of antioxidants and MAP kinase inhibitor. Moreover, astrocytes bearing the amyotrophic lateral sclerosis (ALS) linked to superoxide dismutase 1 G93A (SOD1^G93A^) mutation also induce motoneuron killing in both coculture conditions and through soluble factors found in the culture media [32], [33], [34], [35]. The fact that astrocytes treated with GA and those bearing the SOD1^G93A^ mutation [22], [36] display comparable defects in mitochondrial membrane potential suggests a common intracellular signaling pathway leading to a neurotoxic astrocytic phenotype. Further, the antioxidant FeTCPP and related compounds with antioxidant and peroxynitrite scavenger activities that prevented GA-induced astrocyte mediated toxicity both in vitro and in vivo are also protective in ALS models [37]. Thus, metalloporphyrins appear as potentially useful adjuvant therapeutics in the acute encephalopathic crisis, which could abrogate the triggering of the pathological process in GA-I patients.

Summarizing, our data propose that astrocytes are key players in GA-I onset and progression. While normal astrocytes could buffer GA toxic effects during the presymptomatic stages of the disease, they could become vulnerable to GA when concentrations critically increase. Our results suggest that once damaged by GA, astrocytes proliferate and follow a long term phenotypic change that appears to promote disease progression autonomously from GA levels. Finally, it is conceivable that an acute pharmacological intervention with FeTCPP will prevent astrocyte proliferation and neurotoxicity, thus ameliorating the otherwise ineluctable course of GA-I.

## Materials and Methods

### Chemicals

Dulbecos modified Eagle's medium (DMEM), Neurobasal medium, glutamine, B27, fetal bovine serum (FBS), penicillin/streptomycin, trypsin, Fluoro JadeC (FJC), 5,5′,6,6′-tetrachloro-1,1′,3,3′-tetraethylbenzimidazolylcarbocyanine iodide (JC1), 5-(and-6)-carboxy-2′,7′-difluorodihydrofluorescein diacetate (carboxy-H2DFFDA) and monochlorobimane were purchased from Invitrogen (Carlsbad, CA, USA). Iron porphyrins came from Alexis Biochemical (San Diego, CA, USA). 1,4-diamino-2,3-dicyano-1,4-bis[2-aminophenylthio] butadiene (U0126) was purchased from Cell Signaling Technology (Danvers, MA, USA). Commercial antibodies came from Dako (Carpinteria, CA, USA), Chemicon (Temecula, CA, USA), Sigma (St Louis, MO, USA), Covance (Princeton, NJ, USA), Cell Signalling (Danvers, MA, USA) and Invitrogen. All other chemicals of analytical grade were obtained from Sigma.

### Ethical statement

This study was carried out in strict accordance with the IIBCE Bioethics Committee's requirements (Number of protocol approbation 001/2/2010) and under the current ethical regulations of the National law for animal experimentation N° 18.611 that follows the Guide for the Care and Use of Laboratory Animals of the National Institutes of Health. All surgery was performed under ketamine∶xilacine anesthesia, and all efforts were made to minimize suffering, discomfort or stress. All efforts were also made in order to use the minimal number of animals necessary to produce reliable scientific data.

### Animals

Sprague Dawley rats, breeded at the IIBCE animal house, were maintained with food and water ad libitum, controlled temperature and 12 h light/dark cycle. 5 whole litters of 12 animals each were used for each experimental in vivo condition. Other 5 whole litters were employed for culturing astrocytes or neurons. All experiments were performed at least three times in duplicates or triplicates.

### GA administration and drug treatment

Each littermate was injected intraventricularlly into the Cisterna magna between 12 and 24 hours after birth (P0) with GA (2.5 µmol/g, pH 7.4) or vehicle (phosphate buffered saline (PBS), 0.01 M, pH 7.4). The dose employed (congruent with 2.5 mM GA in body and around 50 mM in cerebrospinal fluid at the moment of administration) was in range with concentrations found in brain of GA-I patients and mimicked the acute crisis they suffer [11], [12], [15]. A maximal volume of 5 µl was injected at 2 µl/min by using 3 mm of an anesthesia 30G needle attached to a Tygon tube extension to allow correct manipulation and zone identification. After injection, animals were allowed to recover at 30°C during 15–30 min and returned to the mother. In some experiments, 5-bromo-2′-deoxyuridine (BrdU, 60 mg/kg, ip) was administered immediately after GA or PBS administration. Treatment with the antioxidant FeTCPP (10–20 µmol/g, ip) was performed before GA or PBS injection.

### Histological processing

After 5 (P5), 12 (P12), 21 (P21) and 45 (P45) days post-injection, 5 animals of each experimental condition were anesthetized with 90 ketamine∶10 xilacine mg/Kg and intracardially perfused with saline and then with 4–10% paraformaldehyde in 0.1 M, pH 7.4 phosphate buffer. After perfusion, brains were quickly removed, postfixed (4 h, 4°C) and maintained in phosphate buffer until sectioning. For each brain, consecutive series of 20 and 50 µm thick coronal sections containing striatal regions were obtained [38], [39] with a Leica 1000S vibratome and either stored free-floating at 4°C or mounted on gelatin-coated slides.

### Immunohistochemistry

For the visualization of astrocytes and/or neurons, parallel free floating sections were processed for the immunohistochemical demonstration of S100β, GFAP (both astroglial markers) or NeuN as a pan-neuronal labeling. Neurofilament and phosphorylated neurofilament staining was made after citrate antigen retrieval as indicated by antibody manufacturers. In all cases, brain sections were washed in PBS (0.01 M, pH 7.4) and incubated with non-specific binding blocking buffer (PBS+0.3% Triton X-100+5% bovine serum albumin or goat serum) for 30 min. Afterwards, sections were incubated overnight at 4°C with pairs of compatible antibodies such as a polyclonal anti-rabbit GFAP (Sigma, 1∶500) together with monoclonal anti-S100β (Sigma, 1∶500), anti-NeuN (Chemicon, 1∶350); or anti-68 kDa (Cell Signaling, 1∶500) or anti-phosphorylated thick neurofilaments (Covance, 1∶1500). After primary antibody incubations, sections were rinsed in PBS, and then incubated at room temperature (21–23°C) for 90 min with either anti-mouse or anti-rabbit Ig conjugated to fluorescent probes (Molecular Probes), both diluted 1∶500–1∶800 in PBS-0.3% Triton. Sections were washed, mounted in glycerol and imaged in a FV300 Olympus confocal microscope. As negative controls, the primary antibodies were omitted.

BrdU was recognized by using a mouse specific antibody (DAKO, 1∶500 in PBS-0.3% Triton X-100 buffered solution, 4°C, and overnight) after HCl denaturation and neutralization [22]. Some BrdU immunostainings were recognized by a secondary antibody conjugated to horse radish peroxidase and diaminobenzidine stain.

Nitrotyrosine recognition was made according to the protocol described above after a heat antigen retrieval as suggested by manufacturers and employing the rabbit anti-nitrotyrosine polyclonal antibody (1∶300, Chemicon).

### Assessment of degenerating neurons

Brain sections were stained with Fluoro-Jade C (FJC) as described by Schmued et al. [40]. Briefly, 20 µm gelatin-sticked dried brain sections were dehydrated-rehydrated, oxidized (0.06% of potassium permanganate) and incubated with 0.0001% FJC at room temperature. After 20 min, sections were washed, dried, cleared in xylene and cover slipped in DPX mounting media. Sections were imaged in a FV300 Olympus confocal microscope using a 488 nm excitation.

### Quantification in brain sections

Anatomical landmarks were used to ensure that parameters were analyzed at similar striatal levels within and between experimental groups [38], [39]. A systematic random sampling was employed and 5 to 11 representative high-power non-overlapping fields covering up to 90% of striatal areas were imaged in 5 to 9 striatal sections of each condition. In each image, all individual nuclei or positive cells were counted manually using the Image J (NIH, USA) cell counter. Total BrdU positive nuclei were counted in striatal sections immunostained for BrdU alone or double labeled with GFAP or S100β. Positive BrdU nuclei were counted in striatal parenchyma once background was measured and corrected to 0. Only the brown dots that duplicated the background values were counted and represented.

The number of FJC positive cells and area of neurofilaments were assessed using the NIH 1.62 and Image J software. In all cases, data obtained in each field per slice were added together providing one value for each slice. Data from all slices per rat were averaged and the final value was used to calculate the relationship between treated and control conditions.

### Cell cultures

Primary astrocyte cultures were prepared from striata of rats aged 1–2 days according to Saneto and De Vellis [41] with minor modifications. Briefly, cultures were obtained from 4–7 striata that were dissociated with trypsin and mechanically, seeded in a proliferating media and enriched in astrocytes by continuous shaking at 37°C during 48 h. Then, astrocytes were plated at 2×10^4^ cells/cm^2^ and maintained in DMEM supplemented with 10% FBS, 3.6 g/l HEPES, 100 IU/ml penicillin and 100 µg/ml streptomycin. Cultures consisted in at least 98% GFAP positive astrocytes and devoid of OX42-positive microglial cells.

Striatal neurons were prepared from E17-18 embryos according to Ventimiglia et al. [42] with minor modifications. Pregnant rats were euthanized and embryos aseptically retired. Striata were removed and received in Neurobasal medium containing 2% B27 and 1 mM glutamine. Tissue was cleaned, minced and dissociated to obtain isolated cells after passing through an 80 µm mesh. Around 300000 cells (3.2–3.4×10^4^ cells/cm^2^ density) were seeded onto plates covered with 0.1 mg/ml poly-D lysine. Half of the media was replaced at day 3 after plating. At 5 days in vitro (DIV) cells were treated with conditioned media for 24 h. In cultured isolated neurons, 97% of living cells were MAP2 positive and GFAP negative. Less than 1% of cells were positive for neuroglican 2.

In co-culture experiments, freshly prepared neurons (around 2×10^4^ cells/cm^2^ density) were seeded on top of confluent astrocyte monolayers previously treated with GA or FeTCPP/GA at 6 hours after withdrawal of the media containing GA and several washes. Evaluation of neuron survival was made 5 days later.

### Astrocyte treatments

Experiments were done in confluence (approximately 1 week after plating). Before each treatment, astrocytes were incubated with DMEM-2% FBS during 24 h and then treated with 5 mM GA (pH 7.4) for 24 h [22]. Appropriate aliquots of a 500 mM acid stock solution, were prepared in 5 N NaOH immediately prior to use to assure neutral pH. In other experiments, cells were pretreated with 20 µM FeTCCP previous to GA addition, media was replaced, cells washed and then GA or PBS was added. Conditioned astrocyte medium was collected 18 h after drug withdrawal and 6 hours of washing. Media collected were spun briefly to obtain a cell-free conditioned medium that was used for treating isolated striatal neurons. Similar protocols were performed by pre-treating astrocytes with 20 µM FeTPPS, 2 µM FeTMPyP, 1 mM apocynin, or 20 µM U0126, during 30 to 60 min previous to add 5 mM GA.

### Assessment of astrocyte mitochondrial potential, glutathione and oxidative levels in living astrocytes

Mitochondrial potential was evaluated as previously described [22]. Briefly, control and GA-treated astrocytes were incubated with the ratiometric dye JC1 (3 µM, 37°C, 15 min, in PBS containing 1 g/L glucose and 0.183 g/L CaCl_2_). The ratio between red and green emission was measured after a single excitation at 488 nm. Glutathione levels were estimated according to Chatterjee et al. [43] by measuring the fluorescence of monochlorobimane-glutathione adducts. Briefly, cells were incubated with monochlorobimane (40 ìM, 37°C, 20 min) and then disrupted with Igepal (0.1% v/v). Emission was measured at 460 nm after a 395 nm excitation. Oxidative activity in control and GA-treated astrocytes was measured with the cell-permeant carboxy-H_2_DFFDA probe. According to manufacturers, living cells were washed and incubated 1 h with 5 µM carboxy-H2DFFDA. Then, 1 µg/ml DAPI was added, cells rinsed and each fluorescence immediately imaged or measured after excitations of 405 and 488 nm, respectively. All data were expressed as percent of respective control values.

### Quantification of survival in cultured neurons

The number of viable neurons was estimated after immunostaining against the neuronal marker microtubule associated protein-2 (MAP-2) or bright field imaging. Briefly, cultures were fixed with −20°C methanol (5 min, 4°C), hydrated for 30 min and blocked with 5% bovine serum albumin in PBS during 60 min at room temperature. Then, cultures were incubated with a monoclonal anti-MAP2 antibody (Chemicon, 1∶400, overnight, 4°C) and with a secondary antibody conjugated to tetramethylrhodamine isothiocyanate (90 min, room temperature). In co-cultures, neurons were recognized by morphology and MAP2 immunoreactivity, whereas astrocytes were identified by positive immunoreactivity against S100β or GFAP (both Sigma, 1∶400). All positive MAP2 cells were counted regardless of its appearance in 90% of whole area seeded. Neuronal survival in isolated cultures was also assessed by the release of lactate dehydrogenase [22].

### Statistical analysis

Statistical analysis of data was performed with Sigma Stat 2.0 using student *t*-test or one-way ANOVA followed by Scheffe post hoc comparison if necessary. All results are presented as mean ± SEM, *p*<0.05 was considered as significant.

## References

[1] Powell EM, Geller HM (1999). Dissection of astrocyte-mediated cues in neuronal guidance and process extension.. Glia.

[2] Barres BA (2008). The mystery and magic of glia: a perspective on their roles in health and disease.. Neuron.

[3] Eroglu C, Barres BA (2010). Regulation of synaptic connectivity by glia.. Nature.

[4] Abbott NJ, Ronnback L, Hansson E (2006). Astrocyte-endothelial interactions at the blood-brain barrier.. Nat Rev Neurosci.

[5] Sullivan SM, Bjorkman ST, Miller SM, Colditz PB, Pow DV (2010). Morphological changes in white matter astrocytes in response to hypoxia/ischemia in the neonatal pig.. Brain Res.

[6] Haynes RL, Folkerth RD, Trachtenberg FL, Volpe JJ, Kinney HC (2009). Nitrosative stress and inducible nitric oxide synthase expression in periventricular leukomalacia.. Acta Neuropathol.

[7] Stolp HB, Ek CJ, Johansson PA, Dziegielewska KM, Bethge N (2009). Factors involved in inflammation-induced developmental white matter damage.. Neurosci Lett.

[8] Sharma R, Fischer MT, Bauer J, Felts PA, Smith KJ (2010). Inflammation induced by innate immunity in the central nervous system leads to primary astrocyte dysfunction followed by demyelination.. Acta Neuropathol.

[9] De Keyser J, Mostert JP, Koch MW (2008). Dysfunctional astrocytes as key players in the pathogenesis of central nervous system disorders.. J Neurol Sci.

[10] Goodman SI, Kratz LE, DiGiulio KA, Biery BJ, Goodman KE (1995). Cloning of glutaryl-CoA dehydrogenase cDNA, and expression of wild type and mutant enzymes in Escherichia coli.. Hum Mol Genet.

[11] Strauss KA, Puffenberger EG, Robinson DL, Morton DH (2003). Type I glutaric aciduria, part 1: natural history of 77 patients.. Am J Med Genet C Semin Med Genet.

[12] Funk CB, Prasad AN, Frosk P, Sauer S, Kolker S (2005). Neuropathological, biochemical and molecular findings in a glutaric acidemia type 1 cohort.. Brain.

[13] Zinnanti WJ, Lazovic J, Housman C, LaNoue K, O'Callaghan JP (2007). Mechanism of age-dependent susceptibility and novel treatment strategy in glutaric acidemia type I.. J Clin Invest.

[14] Sauer SW, Okun JG, Fricker G, Mahringer A, Muller I (2006). Intracerebral accumulation of glutaric and 3-hydroxyglutaric acids secondary to limited flux across the blood-brain barrier constitute a biochemical risk factor for neurodegeneration in glutaryl-CoA dehydrogenase deficiency.. J Neurochem.

[15] Hoffmann GF, Athanassopoulos S, Burlina AB, Duran M, de Klerk JB (1996). Clinical course, early diagnosis, treatment, and prevention of disease in glutaryl-CoA dehydrogenase deficiency.. Neuropediatrics.

[16] Leibel RL, Shih VE, Goodman SI, Bauman ML, McCabe ER (1980). Glutaric acidemia: a metabolic disorder causing progressive choreoathetosis.. Neurology.

[17] Kolker S, Ahlemeyer B, Krieglstein J, Hoffmann GF (2000). Cerebral organic acid disorders induce neuronal damage via excitotoxic organic acids in vitro.. Amino Acids.

[18] Soffer D, Amir N, Elpeleg ON, Gomori JM, Shalev RS (1992). Striatal degeneration and spongy myelinopathy in glutaric acidemia.. J Neurol Sci.

[19] Ferreira Gda C, Viegas CM, Schuck PF, Tonin A, Ribeiro CA (2005). Glutaric acid administration impairs energy metabolism in midbrain and skeletal muscle of young rats.. Neurochem Res.

[20] Latini A, Ferreira GC, Scussiato K, Schuck PF, Solano AF (2007). Induction of oxidative stress by chronic and acute glutaric acid administration to rats.. Cell Mol Neurobiol.

[21] Lamp J, Keyser B, Koeller DM, Ullrich K, Braulke T (2011). Glutaric aciduria type 1 metabolites impair the succinate transport from astrocytic to neuronal cells.. J Biol Chem.

[22] Olivera S, Fernandez A, Latini A, Rosillo JC, Casanova G (2008). Astrocytic proliferation and mitochondrial dysfunction induced by accumulated glutaric acidemia I (GAI) metabolites: possible implications for GAI pathogenesis.. Neurobiol Dis.

[23] Rosa RB, Dalcin KB, Schmidt AL, Gerhardt D, Ribeiro CA (2007). Evidence that glutaric acid reduces glutamate uptake by cerebral cortex of infant rats.. Life Sci.

[24] Cassina P, Peluffo H, Pehar M, Martinez-Palma L, Ressia A (2002). Peroxynitrite triggers a phenotypic transformation in spinal cord astrocytes that induces motor neuron apoptosis.. J Neurosci Res.

[25] Zacharaki T, Sophou S, Giannakopoulou A, Dinopoulos A, Antonopoulos J (2010). Natural and lesion-induced apoptosis in the dorsal lateral geniculate nucleus during development.. Brain Res.

[26] Abramov AY, Duchen MR (2005). The role of an astrocytic NADPH oxidase in the neurotoxicity of amyloid beta peptides.. Philos Trans R Soc Lond B Biol Sci.

[27] Zhu C, Qiu L, Wang X, Xu F, Nilsson M (2009). Age-dependent regenerative responses in the striatum and cortex after hypoxia-ischemia.. J Cereb Blood Flow Metab.

[28] Bain JM, Ziegler A, Yang Z, Levison SW, Sen E (2010). TGFbeta1 stimulates the over-production of white matter astrocytes from precursors of the “brain marrow” in a rodent model of neonatal encephalopathy.. PLoS One.

[29] Kolker S, Sauer SW, Hoffmann GF, Muller I, Morath MA (2008). Pathogenesis of CNS involvement in disorders of amino and organic acid metabolism.. J Inherit Metab Dis.

[30] Brozzi F, Arcuri C, Giambanco I, Donato R (2009). S100B Protein Regulates Astrocyte Shape and Migration via Interaction with Src Kinase: IMPLICATIONS FOR ASTROCYTE DEVELOPMENT, ACTIVATION, AND TUMOR GROWTH.. J Biol Chem.

[31] Donato R, Sorci G, Riuzzi F, Arcuri C, Bianchi R (2009). S100B's double life: intracellular regulator and extracellular signal.. Biochim Biophys Acta.

[32] Di Giorgio FP, Carrasco MA, Siao MC, Maniatis T, Eggan K (2007). Non-cell autonomous effect of glia on motor neurons in an embryonic stem cell-based ALS model.. Nat Neurosci.

[33] Vargas MR, Pehar M, Diaz-Amarilla PJ, Beckman JS, Barbeito L (2008). Transcriptional profile of primary astrocytes expressing ALS-linked mutant SOD1.. J Neurosci Res.

[34] Yamanaka K, Chun SJ, Boillee S, Fujimori-Tonou N, Yamashita H (2008). Astrocytes as determinants of disease progression in inherited amyotrophic lateral sclerosis.. Nat Neurosci.

[35] Barbeito LH, Pehar M, Cassina P, Vargas MR, Peluffo H (2004). A role for astrocytes in motor neuron loss in amyotrophic lateral sclerosis.. Brain Res Brain Res Rev.

[36] Cassina P, Cassina A, Pehar M, Castellanos R, Gandelman M (2008). Mitochondrial dysfunction in SOD1G93A-bearing astrocytes promotes motor neuron degeneration: prevention by mitochondrial-targeted antioxidants.. J Neurosci.

[37] Crow JP, Calingasan NY, Chen J, Hill JL, Beal MF (2005). Manganese porphyrin given at symptom onset markedly extends survival of ALS mice.. Ann Neurol.

[38] Paxinos G, Watson C, Press A (1982). The rat brain in stereotaxic coordinates..

[39] Paxinos G, Törk I, Tecott LH, Valentino KL, Press A (1991). Atlas of developing rat brain..

[40] Schmued LC, Stowers CC, Scallet AC, Xu L (2005). Fluoro-Jade C results in ultra high resolution and contrast labeling of degenerating neurons.. Brain Res.

[41] Saneto RP, Chiappelli F, de Vellis J (1987). Interleukin-2 inhibition of oligodendrocyte progenitor cell proliferation depends on expression of the TAC receptor.. J Neurosci Res.

[42] Ventimiglia R, Mather PE, Jones BE, Lindsay RM (1995). The neurotrophins BDNF, NT-3 and NT-4/5 promote survival and morphological and biochemical differentiation of striatal neurons in vitro.. Eur J Neurosci.

[43] Chatterjee S, Noack H, Possel H, Keilhoff G, Wolf G (1999). Glutathione levels in primary glial cultures: monochlorobimane provides evidence of cell type-specific distribution.. Glia.

